# Fast Single-Image HDR Tone-Mapping by Avoiding Base Layer Extraction

**DOI:** 10.3390/s20164378

**Published:** 2020-08-05

**Authors:** Masud An-Nur Islam Fahim, Ho Yub Jung

**Affiliations:** Department of Computer Engineering, Chosun University, Gwangju 61452, Korea; mostofa21fahim@gmail.com

**Keywords:** contrast stretching, camera response function, tone-mapping, adaptive parameters

## Abstract

The tone-mapping algorithm compresses the high dynamic range (HDR) information into the standard dynamic range for regular devices. An ideal tone-mapping algorithm reproduces the HDR image without losing any vital information. The usual tone-mapping algorithms mostly deal with detail layer enhancement and gradient-domain manipulation with the help of a smoothing operator. However, these approaches often have to face challenges with over enhancement, halo effects, and over-saturation effects. To address these challenges, we propose a two-step solution to perform a tone-mapping operation using contrast enhancement. Our method improves the performance of the camera response model by utilizing the improved adaptive parameter selection and weight matrix extraction. Experiments show that our method performs reasonably well for overexposed and underexposed HDR images without producing any ringing or halo effects.

## 1. Introduction

**Figure 1:**
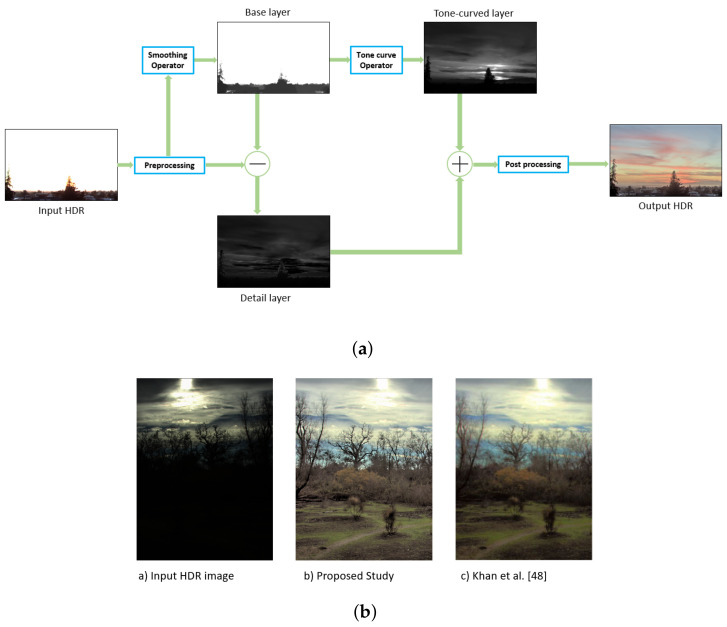
(**a**) Example of the typical approach of tone-mapping procedure. (**b**) Tone mapping performance of the proposed study.

Casual photographic devices can represent a fraction of the HDR images due to their limited irradiance range. That is why HDR images displayed by regular devices often look overexposed or underexposed. Therefore, we need a proper tone-mapping algorithm to compress the HDR data into the standard dynamic range (SDR), which is compatible with regular devices. A typical tone-mapping algorithm tries to transform the HDR image into an SDR image without losing any vital spatial information. An example of this is presented in Figure 1.

In recent years, many studies have been conducted on HDR tone-mapping. Even though a significant amount of variance is present in previous tone-mapping algorithms, many studies propose using a base-and-detail layer decomposition to transform HDR into SDR. In this method, the base layer and the detail layer are extracted with the help of a standard edge-aware smoothing algorithm. Each layer goes through an individual manipulation step and unified together through augmentation, leading to the desired transformed image. This approach can faithfully reconstruct the HDR image as an SDR image based upon the smoothing operator. However, several factors are needed to keep in mind during this procedure. The size of the HDR images is generally more massive than the SDR images. Consequently, smoothing operators take a longer time to extract the detail layer and base layer. Detail enhancement-based methods can increase the aesthetics and luminance stretching of an HDR image, although they have to face some challenges due to this enhancement operation. A usual scenario is excessive texture detail enhancement. It is inherently caused by the prevalent tone-mapping operators being unaware of the spatial properties of the detail layer, which leads to a cartoon-like view of the reconstructed image. Halo artifacts are common in typical tone-mapping algorithms because of the inherent lack of smoothing operators. If the underlying smoothing algorithm cannot provide edge-aware smoothing, halo effects remain in processed images. Apart from the previously mentioned scenarios, the inverse gradient problem may arise due to the over smoothing property of the base layer extractor. To faithfully reconstruct HDR images without any artifacts, one may incorporate relevant priors or a robust edge-aware smoothing operator in the base and detail layer extraction algorithm.

The gradient-based tone-mapping operation has gained more attention in recent years because the gradient’s sensitivity is more tractable to the visual system compared to its absolute form [1]. The gradient-based approach takes the magnitude of the gradient into account for tone-mapping operation. This approach operates on larger gradient values to compress the base layer and smaller gradient values for the structure layer. After these operations, augmentation of all the gradients leads to the tone corrected output [1]. Gradient manipulation is crucial in image enhancement since it aids in simultaneous gradient-based image sharpening and image smoothing [1]. In contrast, it faces an unknown intensity range due to gradient integration for which pixel value exceeds the standard radiance bound. To mitigate the above-mentioned scenario, this type of approach often has to incorporate radiance clipping to keep the tone mapped image into the fixed dynamic range. Further, it requires post-processing operations, which sometimes lead to over-saturation or over smoothing [1].

Herein, we present a computationally light and noise-suppressive method that does not suffer from the contrast problem, display adaptive, artifact suppressive, and independent of parameter tweaking. The underlying reason for these features lies in our perception of the tone-mapping problem. In this study, we propose an approach that does not follow the usual way of gradient-domain operation or base layer extraction. Instead of regarding the tone-mapping problem as a base layer or gradient-domain correction problem, our approach treats this problem as a contrast enhancement problem. Accordingly, we do not have to extract the base layer or the gradient layer, which depends significantly on the smoothing operation. This smoothing operation makes the overall computation heavier, and it gets worse with the size of the input image. Additionally, usual smoothing approaches are not free from parameter dependency and involve a post-processing operation.

In contrast to the traditional tone-mapping approach, we proposed an adaptive camera response function with an appropriate weight matrix operator, which makes our approach independent of parameter selection and any post-processing operation. Usual tone-mapping operation accumulates the detail layer or gradient at the end of their approach. This accumulation increases the overall sharpness of the image, as well as the visual clarity. However, this may also increase the noise, introduce the ringing or halo effect, or might lead to the undesirable saturation of the tone mapped image [1]. Since our study does not rely on this approach, the proposed method does not introduce any halo or ringing effects. Further, this study faithfully approximates the exposure information of the input HDR image. This property helps our method to be more noise-suppressive compared to other methods. In summary, our contributions are as follows:

- Our approach obtains tone-mapped HDR images with the help of contrast enhancement, making it unnecessary to perform any smoothing operations.
- The proposed approach tries to approximate the exposure information of the input HDR image faithfully. This information aids contrast enhancement so that our method does not require any post-processing.
- The proposed adaptive parameter selection improves the holistic contrast correction performance.
- Our utilized weight matrix extraction scheme [2] improves the overall contrast optimization performance.
- Since this approach does not involve a smoothing operation or detail enhancement, tone-mapped images do not exhibit ringing effect or halo effect. Additionally, it is computationally faster than other state-of-the-art methods due to its single-channel contrast optimization step.

The structure of our paper is as follows. Section 2 discusses related studies of tone mapping. Section 3 presents the proposed tone-mapping method. Section 4 presents the experimental results, and Section 5 concludes the paper.

## 2. Related Work

Earlier studies of HDR tone mapping can be classified based on performing local and global tone-mapping operations. Several methods [3,4,5] transform HDR images into LDR images using a global tone-mapping operation. The authors of [3] segment the HDR image into two subsections based upon the irradiance value. Afterward, they apply different logarithmic compressions to each section. Tumblin et al. [4] proposed a global brightness-preserving algorithm for HDR tone mapping. Ward et al. [5] mapped HDR images into SDR images by compressing the contrast instead of the luminance of the input images, using a linear compression function.

Global tone mapping leads to locally distorted tone mapping, which has been addressed in local tone mapping-based study [6]. In [7], an HDR image was divided into 11 local irradiance zones and quantized into a compressed form according to those regions. Ma et al. [8] used optimization to enhance the local region visibility. The researchers designed the tone-mapped image quality index (TMQI) [9] as the objective for their optimization algorithm. Duan et al. [10] performed tone mapping for HDR images by correcting the local histogram. Their approach utilized a global contrast correction in the first stage and implemented the same contrast correction algorithm locally. Sira et al. [11] proposed tone correction with a combination of local and global tone mapping. In the first stage, the researchers corrected the saturation globally based on human perception’s properties. In the second stage, they compressed the tone of the input image locally by using a variational model.

Shan et al. [12] have used local linear adjustments on small overlapping windows for the whole HDR image. In this way, each of the overlapping windows has acted as a guidance map which effectively suppresses the local irradiance anomaly. For tone-mapping, symmetrical analysis-synthesis filter banks have used by Li et al. [13]. Their work has exploited local gain control in each sub-band to achieve adaptive property. Gu et al. [14] introduced a local gamma correction with adaptive parameters as an optimization problem to perform tone mapping for an HDR image. Chen et al. [15] proposed a luminance-driven perceptual grouping process to estimate a sparse representation of an HDR image’s irradiance. Due to sub-grouping, researchers could apply a piece-wise illuminance optimization to suppress excessive irradiance values.

Fattal et al. [16] proposed a gradient-domain optimization scheme to tone map the HDR images. They obtained a low-dynamic-range image by solving the Poisson equation. This study [17] manipulated the gradient domain by using the wavelet operation. With the help of edge-avoiding wavelets, researchers reconstructed the HDR image as an LDR image with common artifacts. Ramesh et al. [18] proposed symmetrically fusing multiple pictures in the gradient domain. Their method can preserve important local perceptual cues and improved temporally coherent contextual features. Another study [19] collected edge spectral information from multi-exposure images and then fused all data into a single image. Afterward, it performed derivative manipulation to produce the enhanced low-dynamic-range image. The fusion-based tone-mapping approach was also used in [20,21].

Durand et al. [22] used a piecewise linear approximation for dissecting the base layer and the detail layer to compress the HDR data. Bo et al. [23] used a locally adaptive edge-preserving filter to perform tone mapping, where the resulting image preserved the salient edges. Meylan et al. [24] proposed a Retinex-based tone-mapping algorithm. The researchers’ method utilized an adaptive filter to protect the high-contrast edges from the artifacts and the principal component analysis to suppress the chromatic distortion. Mai et al. [25] proposed a statistical model that approximated the deviation due to the tone mapping and compression operations. The authors optimized the tone curve based upon that model to perform tone mapping. Neil et al. [26] proposed minimal-bracketing algorithms for computing the minimum-sized exposure to compress an HDR image into an LDR image. Malik et al. [27] combined the fusion of different exposures with film response recovery to create an LDR image. Zeev et al. [28] used the weighted least-squares filter for tone mapping. Even though L1 smoothing is an excellent option for edge-aware filtering, it leads to a weak structural prior. To address this, Liang et al. [29] used the L1-L0 model to render an LDR image from an HDR image.

Choudhury et al. [30] proposed a denoising-based detail enhancing approach for tone mapping, which was slower due to its prepossessing and post-processing operations. A local Laplacian filter [31] is very efficient in suppressing the halo effect; however, the relevant operations are prolonged and introduce unnecessary saturation. Recent tone mapping studies have leaned towards CNN-based approaches due to their efficient performance. This study aims to perform inverse tone mapping operation by correcting the input LDR image’s saturation information by using a convolutional neural network. Then they have used a linear function upon the concatenated LDR and the corrected LDR to reconstruct the HDR image [32]. Instead of using multi-exposure input, this study [33] predicted multi-exposures from the input LDR image. Their CNN scheme was later followed by a stack-based fusion step to reconstruct the tone-mapped HDR image. Their approach can efficiently deal with saturation correction but suffers from the linearization problem.

Marnerides et al. [34] avoided the linearization problem by using CNN to reproduce the HDR image directly. However, their method faces challenges with HDR compression due to their utilized normalization scheme. To tackle this, Yuma et al. [35] proposed the L1-Cosine loss function to reconstruct HDR images successfully. Authors claim that their CNN scheme can learn the non-linear relationship between the input LDR image and the reconstructed HDR image. However, CNN-based studies are not of the datasets. Also, existing datasets out there are not comprise of the evenly distributed objects. On the contrary, mathematical modeling-based approaches can be free of dataset dependency. For this, we sought to propose a scheme that can tone map the HDR images as efficiently as possible.

## 3. Methodology

**Figure 2:**
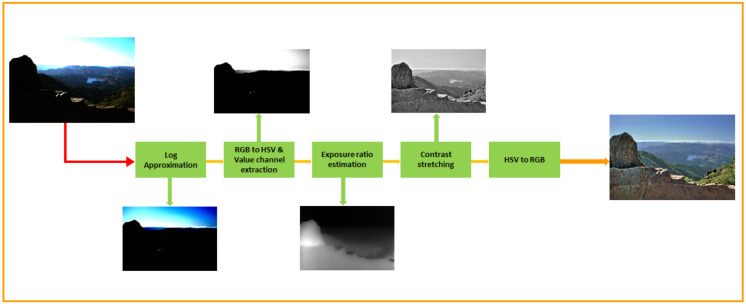
The left-most image is the input image, and the image at the most right position is the tone mapped output image. For ease of view, we have presented the normalized view of the exposure. Here, we estimate the exposure ratio [36] with our proposed adaptive parameter settings, and we have improved the contrast stretching with the help of this weight matrix extraction scheme [2].

In this study, we propose a contrast optimization method to render a high-dynamic-range image as a low-dynamic-range image. A visual representation of our proposed algorithm is present in Figure 2. In the first step, we apply a logarithmic transformation to the input image to bound the irradiance information to a range from 0 to 1. At this stage, we can treat the input image as an LDR image with a reduced contrast distribution. Afterward, we apply an RGB-to-HSV transformation to this image. Next, we extract the value channel and plug that image into an adaptive camera response function to obtain the exposure ratio information of the input HDR image. This information is later used to perform non-linear contrast stretching. In the last stage, we performed an HSV-to-RGB transformation to obtain the tone-mapped HDR image. The entire process does not involve base layer extraction or detail enhancement as do the traditional methods. Therefore, our reconstructed image does not exhibit halo effects, ringing effects, or gradient reversal since the proposed model does not entail gradient-domain operations or base layer extraction.

We can cluster the popular contrast enhancement algorithms into two groups: (1) global contrast enhancement [37,38], and (2) local contrast enhancement [39,40]. The global contrast enhancement technique enhances the image contrast without considering the spatial properties of the input image. Hence, a typical global contrast enhancement algorithm performs a linear contrast enhancement. Due to this, the resulting image contains an overly saturated region or distorted detail. Several studies [41,42,43] have performed nonlinear contrast enhancement to mitigate these challenges. On the other hand, local contrast enhancement techniques prioritize the spatial distribution and achieve better contrast correction, although the several studies did not provide any theoretical justification [13]. Unlike other approaches, the Retinex theory assumes that the internal structure of light decomposes into two parts: (1) an illumination layer, and (2) the scene reflection layer [36]. Popular Retinex studies [36] enhance an input image by manipulating the illumination layer. Since this approach does not consider the camera response properties, it faces the challenges of over- and under-enhancement [1]. These studies [44,45,46,47] combined contrast optimization and detail enhancement to perform the HDR tone mapping and reconstruct HDR images with over saturation and undesirable detail suppression. This study [48] uses global histogram correction for tone mapping.

However, these problems can be alleviated if we can process the image with the proper exposure information. The camera response function tries to mitigate this situation by approximating the exposure information for the input image. If the pixel information captured by the sensors is E and X is the non-linear function which takes E as its input to enhance the contrast, then the output image O is as follows:

(1) O=X(E)

This non-linear function is known as the camera response function. Direct approximation of this function is possible through the ensemble of polynomial model approximation and optimization. However, the nearly accurate estimation of this function is possible through the brightness transformation function (BTF) [36]. If $O^{\prime }$ is the output image and B is the brightness transformation function, for the exposure ratio R, then the desired contrast-corrected approximation of the input image I is as follows:

(2) $$O^{\prime }=B\left(I,R\right)$$

The above equation is also known as the brightness transformation function model [36]. For the above equation, we can write down the camera response function [1,36] for recovering the input HDR image with desired exposure is as follows:

(3) $$O^{\prime }=exp\left(p_{1}\left(1-R^{p_{2}}\right)\right)*I^{R^{p_{2}}}$$

(4) $$\beta =exp\left(p_{1}\left(1-R^{p_{2}}\right)\right),\gamma =R^{p_{2}}$$

**Figure 3:**
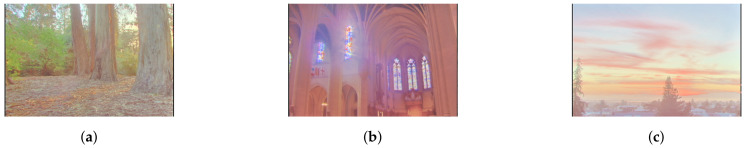
Example of over brightness due to fixed parameters of the camera response function model. For the (**a**–**c**), we can see that overall saturation degrades significantly. Due to over brightness, significant detail loss is present in (**a**,**b**). For (**c**), over-brightness leads to a cartoon-like effect.

Here, $p_{1}$ and $p_{2}$ are model parameters. The default values of $p_{1}$ and $p_{2}$ from the previous study are −0.32 and 1.3 [36]. However, these values lead to over-whitening for several images, as shown in Figure 3. We assessed the effect of parameters experimentally and devised an adaptive form of this model. To determine the values of beta and gamma, we first consider $p_{1}$ and $p_{2}$ to be equal. For any values of $p_{1}$ and $p_{2}$, if the value of gamma is greater than 1, the obtained value of beta will be less than 1. Even though the current parameters are suitable for the brighter region, the rendered image will be darker. The reverse scenario of gamma < 1 and beta >1 will lead to a brighter image.

**Figure 4:**
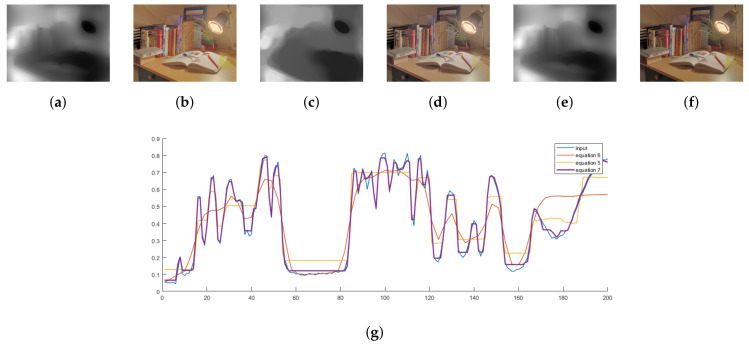
The above figure shows the effects of weight matrices and their respective outputs for the table lamp image. Image (**a**) is the resultant from the Equation (6) produced Image (**b**) with the brightest intensities and vibrant colors. This property contradicts the naturalness of the input scenario. The fourth Image (**d**) is a little dimmer than the first and slightly hazy compared to our Image (**f**). For the shown images, the contrast resulting from the use of Equation (7) causes them to appear more natural. Image (**g**) shows the approximation performance of the weight matrices for a scan line from the input image. For Equation (5), the scan line is least similar to the input scan line, which makes it less aware of the input image’s spatial property. For Equation (7), the resultant scan line is more likely to the input scan line, and from Image (**f**), produced output has a more desirable contrast distribution than Image (**b**) and Image (**d**). (**a**) Equation (6), (**c**) Equation (5), (**e**) Equation (7).

To estimate the values of these parameters adaptively, we first calculate σ, which is the standard deviation of the input image. Next, we set the value of $p_{1}$ to 1 + σ and that of $p_{2}$ to −$p_{1}/4$. We have estimated these parameters by trial-and-error. Our method achieves more accurate color representation than the original parameter values due to this adaptive parameterization. Additionally, avoiding the use of strictly fixed parameters allows us to retain the tone-mapped image’s naturalness. More on the image’s naturalness is present in Figure 4.

**Figure 5:**
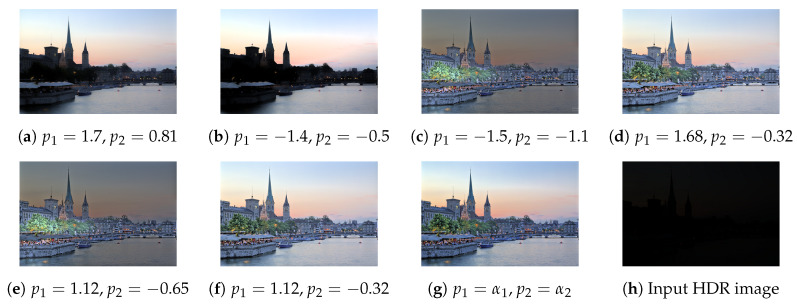
Effect of parameters on tone mapping. The subfigures show the following scenarios:(**a**) *p*_1_ and *p*_2_ increase towards the positive infinity, (**b**) *p*_1_ and *p*_2_ increase in magnitude and tend towards the negative infinity, (**c**) *p*_1_ increases towards the positive infinity, and *p*_2_ tends towards the negative infinity, (**d**) *p*_2_ is fixed, and *p*_1_ increases towards the positive infinity, (**e**) *p*_1_ is fixed, and *p*_2_ tends towards the negative infinity, (**f**) original parameters from [36], (g) proposed parameters *α*_1_ = 1 + *σ*, *α*_2_ = −*α*_1_/4; where *σ* is the standard deviation of the respective image, and (**h**) input HDR image. The tone-mapped image is brighter because of the fixed parameters, and our adaptive settings produce a more realistic image than do the fixed parameter settings.

**Figure 6:**
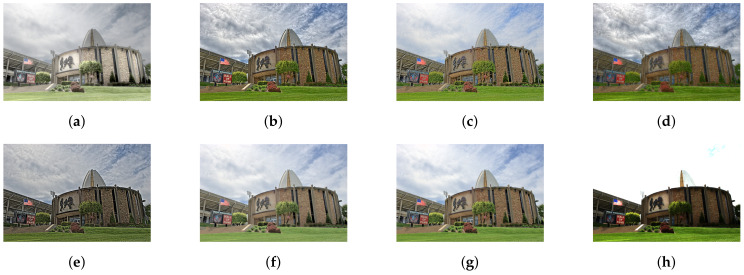
Comparison of tone-mapping methods. (**a**) LW [12], (**b**) WLS [28], (**c**) RoG [2], (**d**) L0 [50], (**e**) IRD [1], (**f**) L0–L1 [29], (**g**) Proposed study, (**h**) Input.

**Figure 7:**
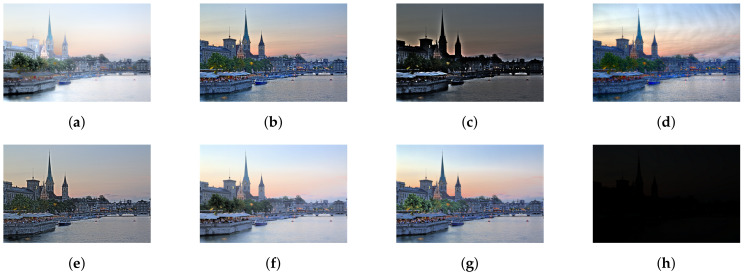
Comparison of tone-mapping methods. (**a**) LW [12], (**b**) WLS [28], (**c**) RoG [2], (**d**) L0 [50], (**e**) IRD [1], (**f**) L0–L1 [29], (**g**) Proposed study, (**h**) Input.

**Figure 8:**
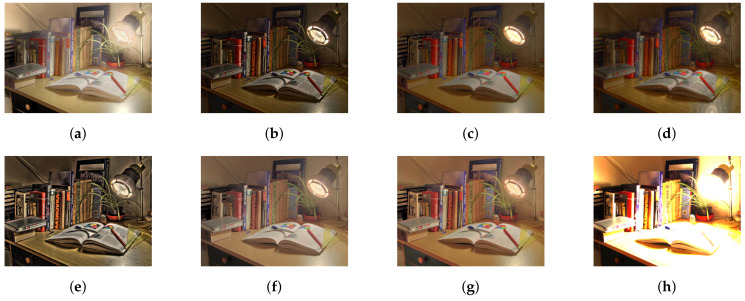
Comparison of tone-mapping methods. (**a**) LW [12], (**b**) WLS [28], (**c**) RoG [2], (**d**) L0 [50], (**e**) IRD [1], (**f**) L0–L1 [29], (**g**) Proposed study, (**h**) Input.

**Figure 9:**
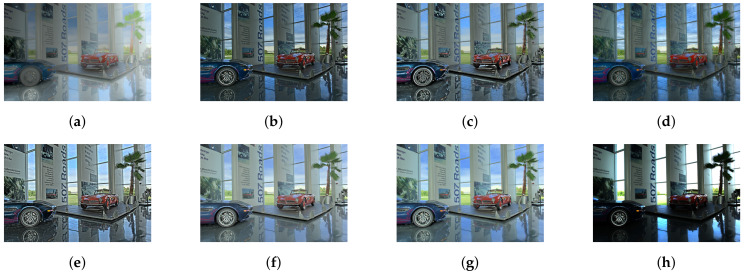
Comparison of tone-mapping methods. (**a**) LW [6], (**b**) WLS [28], (**c**) RoG [2], (**d**) L0 [50], (**e**) IRD [1], (**f**) L0–L1 [29], (**g**) Proposed study, (**h**) Input.

**Figure 10:**
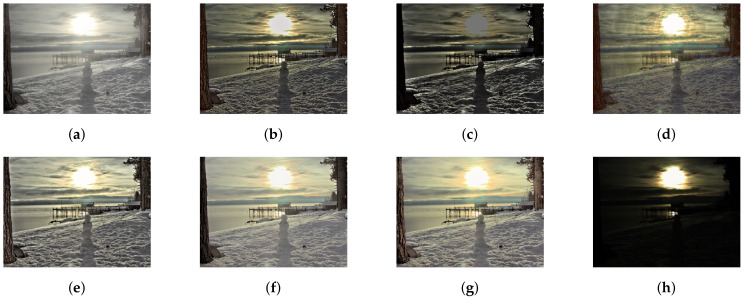
Comparison of tone-mapping methods. (**a**) LW [12], (**b**) WLS [28], (**c**) RoG [2], (**d**) L0 [50], (**e**) IRD [1], (**f**) L0–L1 [29], (**g**) Proposed study, (**h**) Input.

As we see, the Equation (3) is the closed-form solution of Equation (2) bounded by the parameters $p_{1}$ and $p_{2}$. These bounds govern a non-linear relationship with the input values and map the whole image from 0 to 1 without any normalization tasks. However, with previous bounds, it is common to have a reconstructed image that exceeds the 0 to 1 limit and distort the overall contrast quality. The proposed adaptive limits can suppress such distortion. Since these parameters do not maintain a linear relationship with the given image, only empirical evidence can justify its efficacy. Figure 5, Figure 6, Figure 7, Figure 8, Figure 9 and Figure 10 provide empirical evidence to show the efficacy of the proposed adaptive parameter settings.

To achieve the exposure ratio map, we have to calculate the value of the illumination map, and the inverse of the illumination map will give us the desired exposure ratio. For this, we have adopted the iteration free solver [49]. This study [49] has used the weight matrix based upon the relative total variation technique as in Equation (5). The choice of their weight matrix seems to influence in producing brighter output, which is not free from RGB noise [49]. In their study, they have used denoiser as an extension to mitigate this challenge. This additional denoising step makes the overall computation process lengthier. As an improvement, this study [36] has avoided the nominator part of the weight matrix from the Equation (5). They proposed their weight matrix as in the Equation (6) to enhance the contrast of the input image. The resultant illumination map achieved by their study is blurrier compared to the illumination map of [49]. Their study does not exploit denoiser at the end of the enhancement procedure and achieves brighter resolution compared to [49]. The weight matrix $W_{D}\left(\right)$ from [49], ref. [36] expressed as follows:

(5) $$W_{D}\left(m\right)=\frac{1}{|\sum_{y\in \omega (m)}\Delta_{D}L\left(n\right)|+\epsilon };D\in \left(W,H\right)$$

(6) $$W_{D}\left(m\right)=\sum_{y\in \omega (m)}\left[\frac{G_{\sigma }\left(m,n\right)}{|\sum_{y\in w(m)}G_{\sigma }\left(m,n\right)\Delta_{D}L\left(n\right)|+\epsilon }\right]$$

here, L() is the illumination information extracted from the HSV transformation, ω() is the local window, $\Delta_{D}$ is gradient operator, $G_{\sigma }\left(m,n\right)$ is the Gaussian kernel, *D* indicates the dimension, W,H indicates horizontal and vertical axes, ϵ is a very small value in order to avoid zero denominator.Images produced by [49] is dimmer and hazier in contrast [36]. Since their method [36] avoid the denominator, produced images are brighter than [49], which in some cases distort the color and naturalness as in the figure below.

To overcome these challenges, we have used the relativity-of-the- Gaussian [2] weight matrix. The reason of using Relativity-of-the-Gaussian weight matrix lies in its cross-scale smoothing property. Due to this, this operator can capture the small and large scale information compared to other operators. The decomposed formation [2] of this operator is adopted as our weight matrix:

(7) $$W_{D}\left(m\right)=G_{\sigma_{1}/2}*\frac{1}{|\left(G_{\sigma_{1}/2}*\Delta_{D}L\right)\left(G_{\sigma_{1}/2}*\Delta_{D}L\right)|+\epsilon };D\in \left(W,H\right)$$

This weight matrix also helps the optimization technique to keep the gradient regulized. So, the optimization function for the illumination map is as follows:

(8) $${}_{T}^{min}\;\sum_{x}\left({\left(T(x)-L(x)\right)}^{2}+\lambda *\sum_{y\in \omega (m)}\frac{W_{D}\left(x\right)*\Delta_{D}T\left(x\right)}{|\Delta_{D}L\left(x\right)|+\epsilon }\right);D\in \left(W,H\right)$$

here, λ is the balancing factor, x is each entity of the given input. *T* is the illumination information from [36]. This optimization aims to obtain *T* on the basis of value channel *L* from the HSV transformation. We have used 0.001 as the fixed value for λ. Since this equation is in quadratic form, a closed-form solution is available and it can be obtained directly [49]. Now, we can write the Equation [36] for the exposure ratio as follows:

(9) $$R=\frac{1}{maximum(T(x),\epsilon )}$$

The result from the Equation (9) contains desirable exposure ratio information. Now we can plug this exposure ratio into Equation Equation (3) to obtain the contrast-corrected value channel for the tone-mapped HDR image. Originally, Equation Equation (3) works for the RGB input image. In our case, this equation takes I as the value channel of the input HDR image and approximates the value channel with the desired contrast. Later, we perform an HSV-to-RGB conversion to obtain the final tone-mapped HDR result.

Ying et al. [36] performed this enhancement for three channels of the input image. Here, we implement it for only the value channel. This procedure does not degrade the overall hue and saturation information significantly. Moreover, our single-channel operation makes the total computation faster than other studies. More about computational time is present in the next section. Additionally, HSV transformation allows the proposed study to robust contrast correction. Due to this transformation, this scheme escapes chromatic distortion as well as incorrect luminescence approximation.

## 4. Comparative Analysis

We have compared our tone-mapping results with several state-of-the-art studies. Our comparative analysis includes L0-L1 base layer decomposition [29], weighted least square filter [28], Relativity-of-the-Gaussian tone-mapping [2], L0 gradient minimization [50], Intensity range decomposition [1], linear windowed tone-mapping [12]. For comparison, we consider subjective, objective, and time analysis. We maintain the default parameter settings for all tone-mapping operators. Our tone mapping operator uses contrast correction at its core. To demonstrate the contrast-correction performance of our algorithm, we have compared our study with CLAHE [51], CRF [36], LIME [49]. For quantitative evaluation, we have used commonly used evaluation matrices like mean absolute error, PSNR, and SSIM. In the later part of the study, we have presented the necessary tables and figures for comparative analysis.

### 4.1. Dataset

In our study, we have used 150 different HDR images from various sources. We have collected them from various researchers over the internet. Due to the unavailability of the ground truth for the HDR images, we have to perform visual and quantitative comparison for performance analysis. For contrast enhancement performance analysis.we have used Kodak 24 true-color image database [52] and Berkeley image database [52].

### 4.2. Visual Analysis

As mentioned above, we have compared our study with six different state-of-art studies. For a fair comparison, we have not included deep learning-based tone mapping studies. Furthermore, our current study does not concerns video tone mapping. Figure 6, Figure 7, Figure 8, Figure 9 and Figure 10 contain side by side comparison between our study and other mentioned studies. From these figures, we can observe that our study performs well along with other studies. Compared to other studies, our study does not produce any over-enhanced image and free from edge hallucination, over-saturation, and ringing effect.

### 4.3. Subjective Analysis

An image can convey equivocal meanings to its viewers from the aesthetic point of view. Hence, we have performed a subjective analysis based on personal opinion. The selected subjects are 16 individuals, and equal numbers of males and females are present in this group. In our subjective analysis, we have used a casual display (ASUS Monitor) to obtain the mean opinion score for our test images. We have presented HDR images to the participants without any annotations, and there was no ground truth for our test images. Participants in our experiment judged test images based on clarity, contrast, and aesthetics. The mean opinion metric ranges from bad (score = 1) to the excellent ( score = 5). The results of our study have achieved comparable and better scores from the viewers. The mean score and the standard deviation for each method are present in Table 1.

### 4.4. TMQI Analysis

To evaluate our method’s performance, we used the tone map quality index (TMQI) [9]. This metric entails two steps. In the first step, it estimates the structural fidelity and the naturalness score. Afterward, it uses a power function to adjust the computed scores and performs an averaging operation to determine the TMQI score for the input HDR and LDR images. The TMQI score ranges from 0 to 1. Attaining a TMQI score close to 1 indicates that the respective tone-mapping method produces sound tone-mapped output. For our study, we have collected 150 HDR images to create our tone-mapping database. The average TMQI score for our tone-mapping operator is 0.9046. The proposed tone-mapping operator has achieved the highest score of 0.9053. Our method has also attained excellent results in preserving naturalness and fidelity. The average naturalness score is 0.5721, and the fidelity score of our method is 0.8619. A comparative analysis for this study is present in Table 2.

As from Table 2, the proposed study achieves a low fidelity score on average. We know that the fidelity score measures the standard deviation of the given image for various scale sizes in the local domain. In other words, it measures the detail capturing performance of the given tone mapping operator. This mechanism justifies the L0 operator’s highest fidelity score even though it tends to hallucinate images due to its over detail enhancement. On the other hand, our study performs tone mapping without applying detail enhancement, which is the sole reason for our low fidelity score.

### 4.5. Time Analysis

In terms of computational time, our method is significantly faster than other state-of-the-art approaches. In contrast to the trivial tone mapping study, the proposed scheme uses contrast optimization to extract exposure ratio over a single channel. Additionally, this optimization is solvable without any iterations. Altogether, these properties reduce the required computational time. On average, our method takes only 2.6 s to perform the tone mapping operation. The results of our time analysis are presented in Table 3. We have used MATLAB to perform this tone-mapping operation with the AMD-Ryzen 5 2600 processor for all the studies.

### 4.6. Contrast Correction Analysis

**Figure 11:**
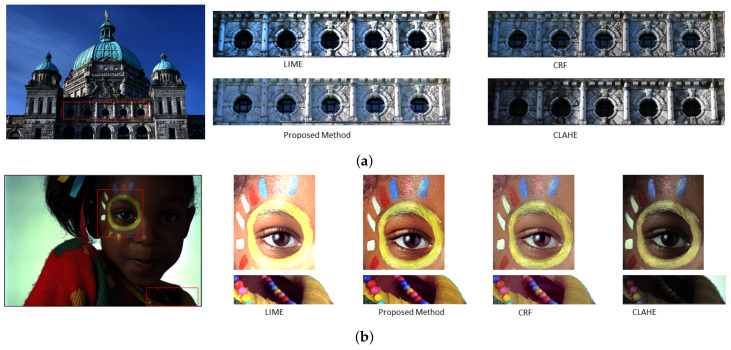
Image contrast enhancement comparison between the proposed method and other studies. (**a**) We select the most cumbersome portion of the image to demonstrate the contrast correction performance. From (**a**), our approach can illuminate the foreground and background image with desirable contrast. (**b**) Contrast performance upon the selected portions from the KODAK-24 true color dataset. We can see that the proposed scheme can illuminate the selected regions without distorting the overall content. On the other hand, compared methods show over contrast stretching.

Our study uses a contrast correcting operator at its heart to tone map the HDR images. Necessarily, the proposed contrast correction method can work well to restore the images with poor contrast. From Figure 11a, the proposed method can enhance the darkest part of the input image without introducing any noise. For the cropped section in Figure 11a, we can see that our study can restore the barely visible hidden tiles. For Figure 11b, we can see that unlike LIME, our method can restore the brightness of the input image without damaging the saturation.

Along with visual performance, proposed approach has demonstrated its efficacy quantitatively, as shown in the Table 4 below. For quantitative analysis, we have estimated the mean absolute error value, SSIM, and PSNR for all the 24 true-color images from the KODAK database [52]. As from the table, we can see that average MAE, PSNR, SSIM achieved by our study outperforms the compared methods.

## 5. Conclusions

In this paper, we have proposed a modified version of the camera response function model (CRFm) for HDR tone-mapping operation. Our proposed adaptive parameter control aids the contrast correction performance of the vanilla CRFm. Additionally, our choice of weight-extracting function helps the camera response function model to maintain the spatial consistency of the input HDR image as well as the low light images. These features altogether improve the visual and physical quality of the tone-mapped images. Our method reduces the tone mapping computational complexity by using only single-channel contrast optimization. Experimental results have shown that without performing the detail enhancement operation, the proposed method can preserve structural fidelity without compromising computational speed and spatial information.

## Figures and Tables

**Table 1:** Subjective evaluation of the compared methods.

Methods	Mean	Standard Deviation
LW [12]	3.46	0.23
WLS [28]	4.1	0.19
RoG [2]	3.2	0.41
L0 [50]	3.0	0.35
IRD [1]	3.68	0.24
L0-L1 [29]	4.46	0.17
Proposed study	**4.51**	0.08

**Table 2:** TMQI evaluation of the compared methods.

Methods	TMQI	Fidelity	Naturalness
LW [12]	0.8616	0.7982	0.4995
WLS [28]	0.8571	0.8578	0.4815
RoG [2]	0.8545	0.8689	0.5037
L0 [50]	0.8679	**0.8704**	0.5122
IRD [1]	0.8713	0.8636	0.5205
L0-L1 [29]	0.8783	0.8423	0.5669
Proposed study	**0.9046**	0.8619	**0.5721**

**Table 3:** Time analysis between the compared methods.

Methods	Time
LW [12]	30.73 s
WLS [28]	10.16 s
RoG [2]	41.2 s
L0 [50]	53.04 s
IRD [1]	78.25 s
L0-L1 [29]	8.73 s
Proposed study	**2.6 s**

**Table 4:** Contrast performance between the compared methods.

Methods	MAE	SSIM	PSNR
LIME [49]	0.0386	0.8512	36.14
CRF [36]	0.0344	0.869	37.55
CLAHE [51]	0.0739	0.783	32.07
Proposed study	**0.0216**	**0.882**	**38.71**
